# Rate‐dependent and unidirectional conduction block between the left pulmonary vein and left atrium after catheter ablation for atrial fibrillation

**DOI:** 10.1002/joa3.12425

**Published:** 2020-09-10

**Authors:** Maki Oi, Shinnosuke Nomura, Mitsunori Miho, Takayasu Kobayashi, Marie Okabayashi, Hirooki Higami, Naoaki Onishi, Nobuya Higashitani, Sayaka Saijo, Fumiko Nakazeki, Naofumi Oyamada, Toshikazu Jinnai, Shohei Terada, Shota Osaki, Katsutoshi Horii, Kazuaki Kaitani

**Affiliations:** ^1^ Department of Cardiovascular Medicine Japanese Red Cross Otsu Hospital Otsu Japan; ^2^ Department of Medical Engineering Japanese Red Cross Otsu Hospital Otsu Japan

**Keywords:** atrial fibrillation, catheter ablation, pulmonary vein isolation, rate‐dependent conduction, unidirectional conduction

## Abstract

A 77‐year‐old woman with symptomatic paroxysmal atrial fibrillation (PAF) underwent pulmonary vein isolation (PVI), but subsequently experienced recurrence. In the second session, unidirectional left atrium (LA)‐left superior pulmonary vein (LSPV) conduction was revealed to exist at the carina of the LSPV. Left pulmonary vein (LPV) pacing performed in a cycle between 300 and 260 ms revealed rate‐dependent pulmonary vein (PV)‐LA conduction, and the location was estimated to be in the roof of the LSPV. PV isolation was achieved after ablation of two gaps. Consideration of the presence of rate‐dependent gaps may be useful to confirm bidirectional block lines after ablation.

## Graphical abstract

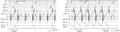

## 1 CASE

**FIGURE 1:**
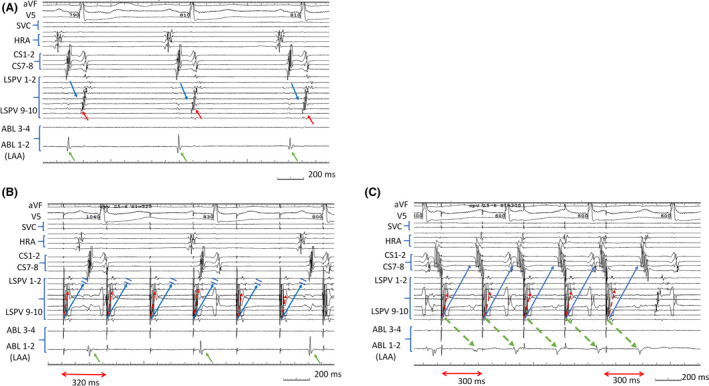
(A) Sinus rhythm shows conduction to the LPV (red arrow). (B) Pacing from the LPV at a pacing cycle length of 320 ms captured local myocardium (red broken arrow) without conducting to the LA (blue arrow) and LAA (green arrow). (C) PV‐LA conduction was revealed when pacing cycle length was shortened to 300 ms. This pacing captured the myocardium of the LSPV (red broken arrow) and was conducted to the LA (blue arrow) and LAA (green broken arrow). LPV, left pulmonary vein; LA, left atrium; LAA, left atrial appendage

**FIGURE 2:**
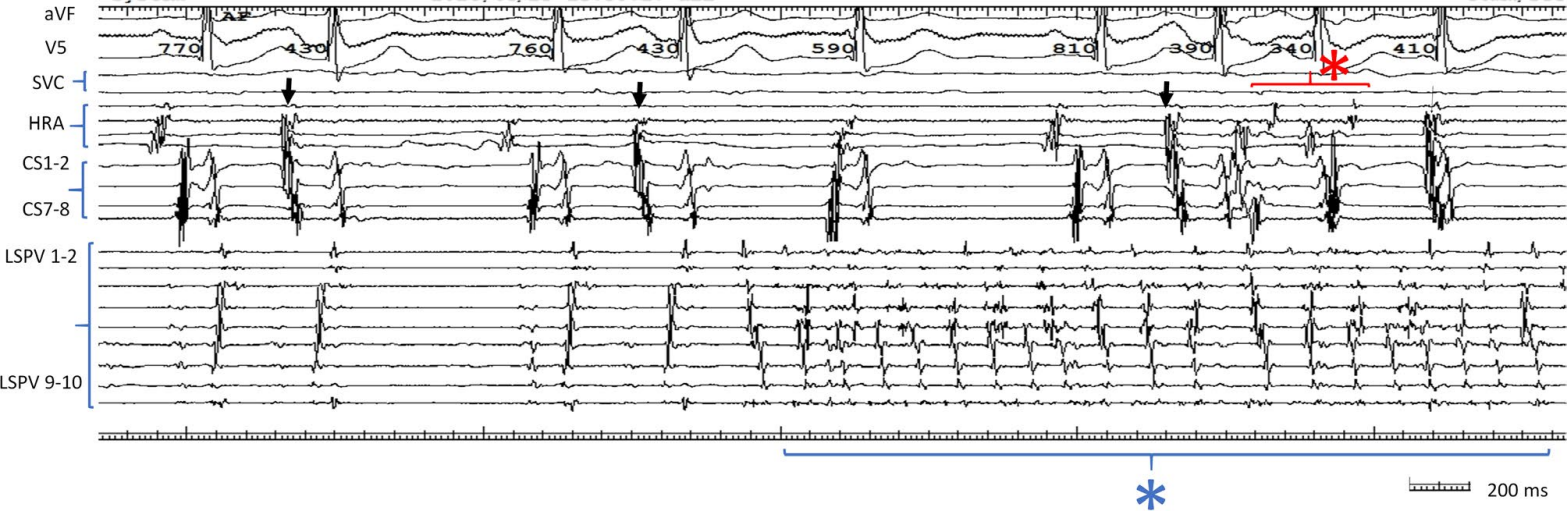
PAC with the same atrial sequence was observed repeatedly (black arrow). Note that the atrial sequence of this PAC was the same as the atrial sequence observed during the pacing of LSPV. Under isoproterenol loading, firing of the LPV under sinus rhythm occurred repeatedly following this PAC (blue asterisk), and this PAC also appeared during this PV firing. After the PAC under PV firing, atrial firing was also observed (red asterisk). LPV, left pulmonary vein; LA, left atrium; PAC, premature atrial contraction

**FIGURE 3:**
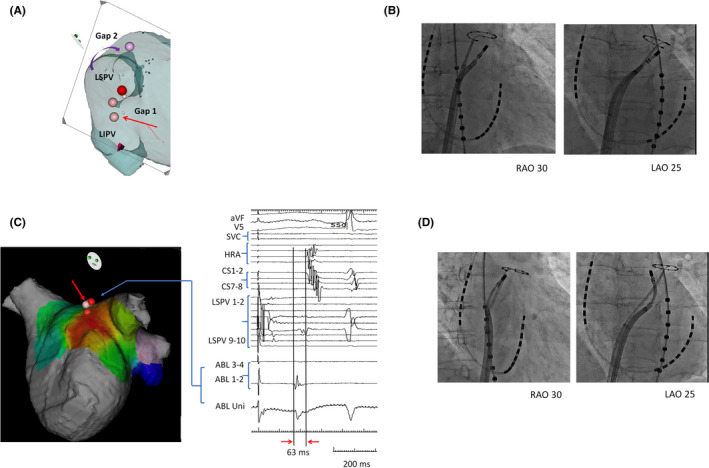
(A) Gap 1 was assumed to exist in the anterior of the carina of the LPV and show LA‐LPV unidirectional conduction. Gap 2 was assumed to exist in the roof of the LSPV and show LPV‐LA unidirectional conduction. Gap 2 was also assumed to have rate‐dependent conduction. (B) Ablation was performed for Gap 1 identified in the anterior carina of the LPV. After ablation of Gap 1, unidirectional LA‐LPV conduction was eliminated (C) Three‐dimensional (3D) electroanatomic activation mapping of LA by CARTO was performed during pacing of the LPV with a cycle length of 260 ms. Gap 2 was revealed as the earliest site of LA activation and was detected in the roof of the LPV (red arrow). Visitags are displayed on the ablation site of Gap 2. The electrogram shows the earliest activation site of the LA in the ablation catheter detected by CARTO mapping during LSPV pacing. This precedes the earliest activation site of the catheter deployed at CS and HRA by 63 ms. (D) Radioscopic image of ablation of Gap 2. After ablation of Gap 2, rate‐dependent LPV‐LA conduction disappears. LPV, left pulmonary vein; LA, left atrium

A 77‐year‐old woman with symptomatic paroxysmal AF (PAF) underwent bilateral enlarged pulmonary vein isolation (PVI) including the left atrial posterior wall. Bilateral conduction block was confirmed during an ATP provocation test and isoproterenol injection. However, recurrence was observed 3 months later and a second ablation session was performed. Both sessions were performed with an 8 Fr irrigated ablation catheter (Thermocool; Biosense Webster, Diamond Bar, CA) connected to the CARTO3 electroanatomic mapping system (Biosense Webster) guided by a target force‐time index of 500 and inter‐lesion distance ≤4 mm. The duration was set as 30 seconds. A 10 pole circular mapping catheter (Lasso; Biosense Webster) was positioned within the pulmonary vein (PV). A 20‐pole electrode catheter (BeeAT; Japan Lifeline, Tokyo) was positioned within the coronary sinus (CS). Bidirectional block was confirmed between the right pulmonary vein (RPV) and left atrium (LA). Subsequently, PV potential was observed in the left superior PV (LSPV) and left inferior PV (LIPV) (Figure 1A). Constant pacing from the Lasso catheter located in the LSPV was performed while shortening the cycle length of stimulation (10 mV, 1.0 ms). Exit block was observed at a pacing cycle length longer than 300 ms (Figure 1B). However, PV‐LA conduction was revealed when pacing cycle length was shortened to 300 ms and continued at a pacing cycle length between 300 and 260 ms (Figure 1C). Exit block reappeared when pacing cycle length was shortened to less than 260 ms. In addition, premature atrial contraction (PAC) with the same atrial sequence as the atrial sequence formed by LSPV pacing was repeatedly observed. After isoproterenol loading, LPV firing occurred repeatedly following this PAC. This PAC also appeared during PV firing inside the pulmonary vein (Figure 2). Two gaps between the PV and LA were speculated: one gap showed unidirectional LA‐LPV conduction located in the anterior of the carina of the LPV (Gap 1), and the other showed unidirectional LPV‐LA and rate‐dependent conduction located in the roof of the LSPV from the atrial sequence (Gap 2) (Figure 3A).

Ablation of Gap 1 was performed and LA‐PV unidirectional conduction disappeared (Figure 3B). However, rate‐dependent LPV‐LA conduction remained after ablation of Gap 1 and the sequence of the atrium formed by pulmonary vein pacing was exactly the same as that observed before the ablation of Gap 1. To identify the location of Gap 2, LA mapping was performed during pacing of the PV with the cycle length at 260 ms. The earliest atrium activation site of the LA was found on the roof of the LSPV (Figure 3C,D). After ablation of Gap 2, rate‐dependent PV‐LA conduction disappeared, and bidirectional block was confirmed during both adenosine and isoproterenol challenge tests. As of 1.5 years later, no recurrence of tachycardia has been confirmed.

## 2 DISCUSSION

We encountered a case with recurrence after AF ablation involving two unidirectional gaps, one of which showed rate‐dependent conduction. Reports of unidirectional rate‐dependent PV‐LA/LA‐PV conduction are very rare. The mechanism underlying rate‐dependent conduction was assumed to be based on phase 4 block. Phase 4 block is a phenomenon often observed in pathological bundle of His‐Purkinje cells, because the slope of phase 4 shows that diastolic depolarization is large and the membrane potential is sufficiently depolarized in the resting phase, resulting in more proximal blockage of excitation. Phase 4 block is triggered by bradycardia, premature contractions, cessation of tachycardia, and long connective periods. Moreover, bradycardia‐dependent conduction blocks can occur without phase 4 block. This is because stimulation of the tachycardia causes a change in the inward current of the cardiomyocyte membrane, depending on the frequency of stimulation, and an action potential is generated in isolated PV tissue.

In this session, PAC with uniform atrial sequence was repeatedly observed (Figure 1D). Sinus rhythm was assumed to be conducted to the LPV via Gap 1, the myocardium of the LPV was captured and was conducted to the LA via Gap 2, forming this PAC. Moreover, the PAC was conducted to the LPV via Gap 1 again. This irregular depolarization of LPV tissue was assumed to trigger the firing inside the LPV. High‐frequency stimulation of tachycardia was reported to cause an action potential in isolated PV tissue. Sustained PV firing might change the conductivity of Gap 2, resulting in AF recurrence. The ATP test is effective to unmask the concealed gap for recurrent cases after AF ablation. If the ATP test is performed before the procedure, LPV‐LA conduction might be revealed.

## CONFLICT OF INTEREST

The authors declare no conflict of interests for this article.

